# Construction of Novel Thermostable Chimeric Vaccine Candidates for Genotype VII Newcastle Disease Virus

**DOI:** 10.3390/v15010082

**Published:** 2022-12-28

**Authors:** Yongzhong Cao, Zongyi Bo, Baoyang Ruan, Mengjiao Guo, Chengcheng Zhang, Xiaorong Zhang, Yantao Wu

**Affiliations:** 1 Joint International Research Laboratory of Agriculture and Agri-Product Safety, The Ministry of Education of China, Yangzhou University, Yangzhou 225009, China; 2 Jiangsu Co-Innovation Center for the Prevention and Control of Animal Infectious Disease and Zoonoses, College of Veterinary Medicine, Yangzhou University, Yangzhou 225009, China

**Keywords:** Newcastle Disease Virus, thermostability, genotype VII-matched chimeric virus, F protein, protection efficiency, viral shedding

## Abstract

Genotype VII Newcastle Disease Virus (NDV) has caused a pandemic in many countries and usually causes fatal consequences in infected chickens. Although current commercial attenuated NDV vaccines can provide an ideal protection against genotype VII NDV, they cannot completely prevent the infection and viral shedding, and the genotype of some vaccine strains cannot match with the prevalent strain. In this study, in order to construct a thermostable and genotype VII-matched live attenuated vaccine, we used a thermostable genotype VIII virulent HR09 strain as the backbone and replaced its F gene with that of the genotype VII DT-2014 strain. Meanwhile, the cleavage site of F gene of DT-2014 was mutated to that of class I F protein and avirulent class II F protein, respectively. The results showed that the two chimeric viruses, designated rcHR09-CI and rcHR09-CII, shared a similar growth kinetics and thermostability with their parental HR09 strain. Mean death time (MDT) and intracerebral pathogenicity index (ICPI) tests showed that the two chimeric viruses were highly attenuated. Though both chimeric NDVs and La Sota vaccine strain could provide complete protection to immunized chickens against the challenge of virulent genotype VII ZJ1 strain, the two chimeric NDVs could induce a higher level of antibody response against ZJ1 strain and could significantly reduce the viral shedding compared with La Sota vaccine strain. In conclusion, our study constructed two chimeric thermostable genotype VII-matched NDV vaccine candidates, which provided complete protection against the challenge of virulent genotype VII NDV.

## 1. Introduction

Newcastle Disease Virus (NDV), the causative agent of Newcastle Disease (ND), belongs to the genus *Orthoavulavirus* in the subfamily Avulavirinae of the family Paramyxoviridae [1]. NDV has a negative-sense, single-stranded RNA genome, which encodes nucleoprotein (N), phosphoprotein (P), matrix protein (M), fusion protein (F), hemagglutininneuraminidase protein (HN), and large protein (L) [2]. Based on the evolution of NDV strains, NDV can be divided into two classes: one is class I viruses, with a genome size of 15,198 nucleotides; another one is class II viruses, with a genome size of 15,186 or 15,192 [3]. Based on its pathogenicity, NDV has been classified into three pathotypes: lentogenic, mesogenic, and velogenic [4]. NDV can be classified into two classes, one is class I NDV which is mainly isolated from wild birds and is always avirulent to chickens. The other is class II NDV, which has been found in domestic and wild birds, including both virulent and avirulent strains [1,5,6].

The F protein of NDV is firstly synthesized as an inactive precursor, F0, which is further cleaved into two active F1 and F2 subunits by host cellular protease [7]. The active F protein can mediate the fusion of viral and cellular membranes [8]. Researchers have found that the cleavage site motif of F protein is also an indicator of the virulence of NDV. It was reported that the virulence strain for chickens showed cleavage site motif of ^112^R/K-R-Q-K/R-R↓F^117^, whereas low virulence NDVs have the motif of ^112^G/E-K/R-QG/E-R↓L^117^ in the same region [9,10]. Therefore, altering the cleavage site motif of F protein is an efficient way to attenuate the virulent NDV [11,12]. Despite its important role in the virulence of NDV, the F protein is also a major contributor to protective immunity of genotype-matched vaccine [13]. 

Thermostable virus vaccine can facilitate its storage and transport, especially for some remote districts where cold chain transportation is not available. Previously, a natural thermostable NDV strain, designated HR09, was isolated in our lab and could keep infectious for more than one hour under 56 °C treatment [14]. Thereafter, using a reverse genetic system, it was found that the residues 315 and 369 in HN protein were responsible for its heat-resistance ability [15]. As HR09 is a virulent strain, we mutated its cleavage site of F protein from ^112^RRQKR↓F^117^ to an avirulent motif ^112^GRQGR↓L^117^ using a reverse genetic system and found that the recombinant virus rHR09 could provide complete protection against the challenge of genotype VII NDV virulent strain [16]. Moreover, the attenuated HR09 was further used as a vaccine vector to express the HA gene of H9N2 subtype avian influenza virus (AIV), which also provided ideal protection to the challenge of H9N2 AIV [17].

In this study, to construct a thermostable and genotype VII-matched NDV attenuated vaccine, we used a thermostable genotype VIII NDV virulent HR09 strain as the backbone and replaced its F gene with that of the genotype VII DT-2014 strain. Meanwhile, the cleavage site of F gene of DT-2014 was mutated to that of class I NDV F protein and avirulent class II NDV F protein, respectively. The results showed the two chimeric viruses shared a similar growth kinetics and thermostability with the HR09. In addition, the mean death time (MDT) and intracerebral pathogenicity index (ICPI) tests showed that the two chimeric viruses belonged to an avirulent strain. Both of them could induce a high level of antibody response against themselves and genotype VII ZJ1 strain in immunized chickens, could significantly decrease viral shedding, and provide full protection against the challenged with ZJ1 strain. In conclusion, our study provides two chimeric thermostable vaccine candidates for genotype VII NDV.

## 2. Materials and Methods

### 2.1. Cells and Viruses

BSR-T7/5 cells, a selective cell line from BHK cells which can express the T7 RNA polymerase, are cultured with Dulbecco’s modified Eagle medium (DMEM, Gibco) supplemented with 15% fetal bovine serum (Pan Biotech, Germany) and 1% penicillin-streptomycin (Thermo Fisher Scientific, Waltham, USA) at 37 °C in the 5% CO_2_ incubator. Specific pathogen free (SPF) embryonated chicken eggs, which can be used for the propagation of NDV, are purchased from Beijing Boehringer Ingelheim Vital Biotechnology Co., Ltd. Thermostable genotype VIII NDV HR09 strain (MF285077.1) and genotype VII ZJ1 strain are stocked in our lab. 

### 2.2. Plasmid Construction

The plasmid, designated pNDV/rHR09, was constructed by cloning the full-length genome sequence of genotype VIII HR09 with the mutation in F protein into TVT7R (0.0) vector [16]. The F gene of genotype VII DT-2014 strain, which had a high similarity with the prevalent genotype VII NDV strains, was amplified, and the mutations of its cleavage sites were generated via site-directed mutagenesis. Then, the F gene in pNDV/rHR09 was replaced by the mutated DT-2014 F gene. All primers sequences used in the study are available upon request. 

### 2.3. Rescue of Recombinant Virus

In this study, two chimeric NDV vaccine candidates were rescued as described previously [18]. The main procedures are described below: firstly, the full-length NDV plasmids which contained the cleavage sites mutated F gene of NDV DT-2014, designated pNDV/rcHR09-CI and pNDV/rcHR09-CII, were transfected with the three helper plasmids pCI-NP, pCI-P, and pCI-L by using Lipofectamine 3000 reagent (Thermo Fisher Scientific, USA) according to the manufacturer’s instruction. Secondly, the allantoic fluids from SPF embryonated chicken eggs were added at 12 h and 36 h post transfection, respectively. Thirdly, the whole cell lysates were collected at 72 h post transfection and set into three times freeze/thawing. Finally, the cell lysates were injected into 10-day-old SPF embryonated chicken eggs for 3–5 days, and the allantoic fluids were collected and subjected into HA assay for the existence of rescued chimeric NDVs.

### 2.4. RT-PCR and QRT-PCR

The RNA was extracted using TRIzol reagent (Merck, Darmstadt, Germany) from the allantoic fluids collected from infected embryonated chicken eggs, and then reverse transcribed into cDNA by using the PrimeScript™ 1st Strand cDNA Synthesis Kit (Takara, Beijing, China). The full-length of rescued NDV genome sequence was amplified using 10 pair primers (available upon request). The levels of virus shedding were quantified by absolute quantitative real-time RT-PCR using a Life Technology instrument. Briefly, the cloacal swab and tracheal swab samples were collected at 3- and 5-days post challenge (dpc), then the RNA was extracted, reverse transcribed to cDNA, and subjected into quantitative real-time PCR (QRT-PCR) for viral shedding quantification. The amount of viral shedding was calculated as the mean ± SD of the mRNA level of all the collected swabs. The primer and Probe sequences used in viral shedding detection are available upon request.

### 2.5. Viral Growth Curve

The growth kinetics of different NDVs were measured in 10-day-old embryonated chicken eggs. Briefly, the NDVs were diluted and injected to 10-day-old embryonated chicken eggs by allantoic inoculation. The allantoic fluid was collected at 24-, 48-, 72-, and 96-h post infection. The EID_50_ of each sample was calculated using the Reed and Muench method [19]. 

### 2.6. Thermostability Test

The thermostability of each virus was measured as previously described [18]. Briefly, each virus with 10^8^ EID_50_ titer at 0.1 mL per vial was subjected into a water bath with 56 °C, then the samples were collected at 10-, 20-, 30-, 40-,50-, and 60-min post incubation. Finally, the virus titer was measured using EID_50_ method.

### 2.7. Virulence and Stability Determination

The virulence of each virus was assessed by applying the mean death time (MDT) test in 10-day-old SPF embryonated chicken eggs as described previously [20,21]. Briefly, the collected allantoic fluid of each chimeric NDV strain was diluted from 10^−6^ to 10^−9^ in 10-fold series and injected into 10-day-old SPF embryonated chicken eggs with 5 per group were incubated with 100 L of each dilution. Then, the challenged embryonated chicken eggs were incubated at 37 °C and candled for total 7 days. The death of each group was recorded, and the MDT value was calculated. In addition, the ICPI test in 1-day-old SPF chickens was also measured to check the virulence of chimeric virus. Briefly, the chimeric virus was diluted in 10-fold series using PBS and injected into 1-day-old SPF embryonated chicken eggs. The incubated eggs were candled for total 8 days and the ICPI value was calculated.

To assess the stability of the two chimeric NDVs, they were propagated in 10-day-old SPF embryonated chicken eggs via allantoic inoculation for total 20 passages, then the MDT and ICPI values of selected passages were measured.

### 2.8. Chicken Immunization and Challenge

A total of 48 8-day-old SPF chickens were randomly divided into four groups, and three groups were inoculated with 10^6^ EID_50_ of rcHR09-CI, rcHR09-CII, and La Sota strain in 100 μL DMEM via the oculonasal route, respectively. The chickens in the control group were injected with 100 μL DMEM. The serum of immunized chickens was collected at 7-, 14-, and 21-days post immunization (dpi) for antibody titer determination. At 21 dpi, the chickens were challenged with 10^5^ EID_50_ NDV genotype VII ZJ1 strain via oculonasal route. The cloacal and oropharyngeal swabs were collected at the 3- and 5- days post challenge (dpc) for further viral shedding detection. The clinical signs and death were recorded every day until 14 dpc.

### 2.9. Serum Antibody Titer Determination

The antibody titer against NDV was measured by hemagglutination inhibition (HI) method as described previously [22].

### 2.10. Ethics Statement

The immunization and challenge of the chickens were approved by the Institutional Animal Care and Use Committee of Yangzhou University (No. YZUDWLL-201904-002; Date: 4 February 2019).

### 2.11. Statistical Analysis

The data are shown as the means ± standard deviations (SDs), and they were obtained from three replicates. The statistical significance between different groups were analyzed by Student’s *t*-test method using GraphPad Prism 7.0 software (La Jolla, CA, USA). (* *p* < 0.05; ** *p* < 0.01; *** *p* < 0.001).

## 3. Results

### 3.1. Schematic for the Construction of Two Chimeric NDVs

**Figure 1:**
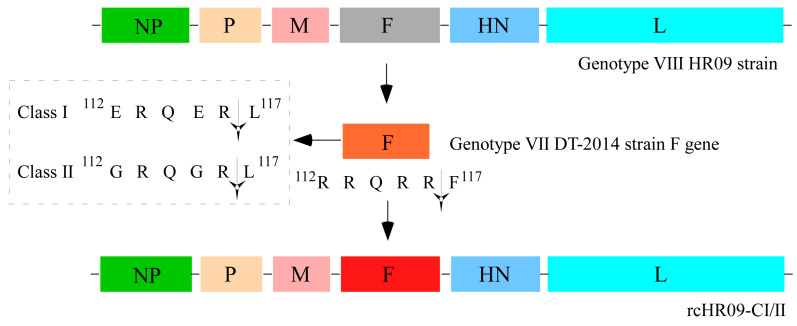
Schematic strategy for the construction of two chimeric NDVs. The F gene of genotype VIII thermostable HR09 strain was replaced with the mutated F gene of genotype VII DT-2014 strain.

Genotype VII NDV has distributed worldwide, and often causes fatal consequences to chickens and other susceptible birds [23]. Previously, we isolated a thermostable genotype VIII NDV HR09 strain, which can maintain infectiousness for more than 60 min when it is incubated at 56 °C [14]. In this study, in order to generate a thermostable attenuated vaccine candidate against NDV genotype VII, we used HR09 as the backbone and replaced the F gene with that of VII DT-2014 strain; meanwhile, the cleavage sites of it were mutated from ^112^RRQRR↓F^117^ to class I NDV F protein cleavage sites ^112^ERQER↓L^117^ and the avirulent strain of class II NDV F protein cleavage sites ^112^GRQGR↓L^117^. The plasmids which contain full-length of HR09 genome with the mutated F protein were designated as prcHR09-CI and prcHR09-CII, respectively (Figure 1).

### 3.2. Construction of the Chimeric NDV rcHR09-CΙ and rcHR09-CⅡ

**Figure 2:**
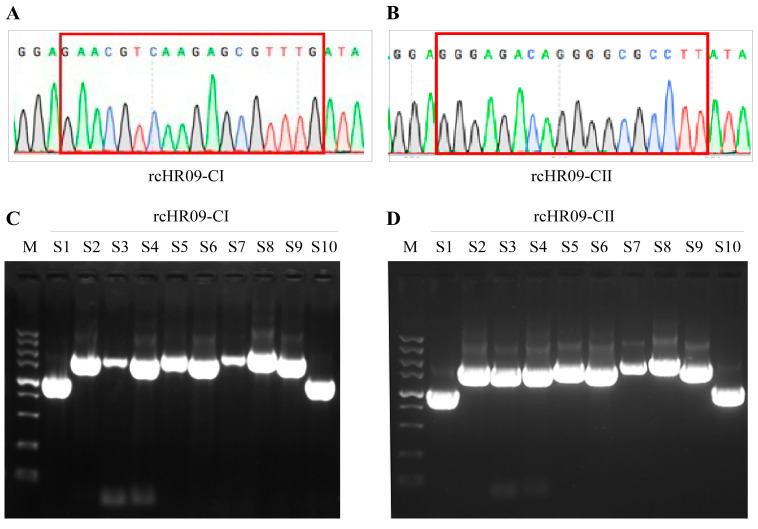
Construction and validation of the chimeric NDV rcHR09-CI and rcHR09-CII. The total RNA of rescued viruses was extracted and reverse-transcribed into cDNA, the F gene of rcHR09-CI (**A**) and rcHR09-CII (**B**) was amplified and set to sequencing. The full length of the chimeric NDV rcHR09-CI (**C**) and rcHR09-CII (**D**) was amplified using 10 pairs of primers, and S1–S10 are the representation of them.

To construct the chimeric viruses, the plasmids prcHR09-CI or prcHR09-CII were co-transfected with the three helper plasmids, pCI-NP, pCI-P, and pCI-L into BSR-T7/5 cells, which can express T7 RNA polymerase. 72 h later, the whole cells lysate was harvested and injected into 10-day-old SPF embryonated eggs, and the allantoic fluids were collected at 4 days post incubation. To check cleavage sites of the two rescued chimeric viruses, the PCR experiment of F gene was performed and the products were subjected to sequencing; the results showed that the cleavage sites were successfully mutated to ^112^ERQER↓L^117^ in rcHR09-CI (Figure 2A) and ^112^GRQGR↓L^117^ in rcHR09-CII (Figure 2B), separately. Finally, the full-length genome of the two thermostable chimeric NDVs were amplified using 10 pairs of primers, and the results demonstrated both rcHR09-CI (Figure 2C) and rcHR09-CII (Figure 2D) contained the full-length genome of NDV. Collectively, these data demonstrated that the two thermostable chimeric viruses were successfully constructed. 

### 3.3. Biological Characteristics of the Chimeric NDV rcHR09-CI and rcHR09-CII

**Figure 3:**
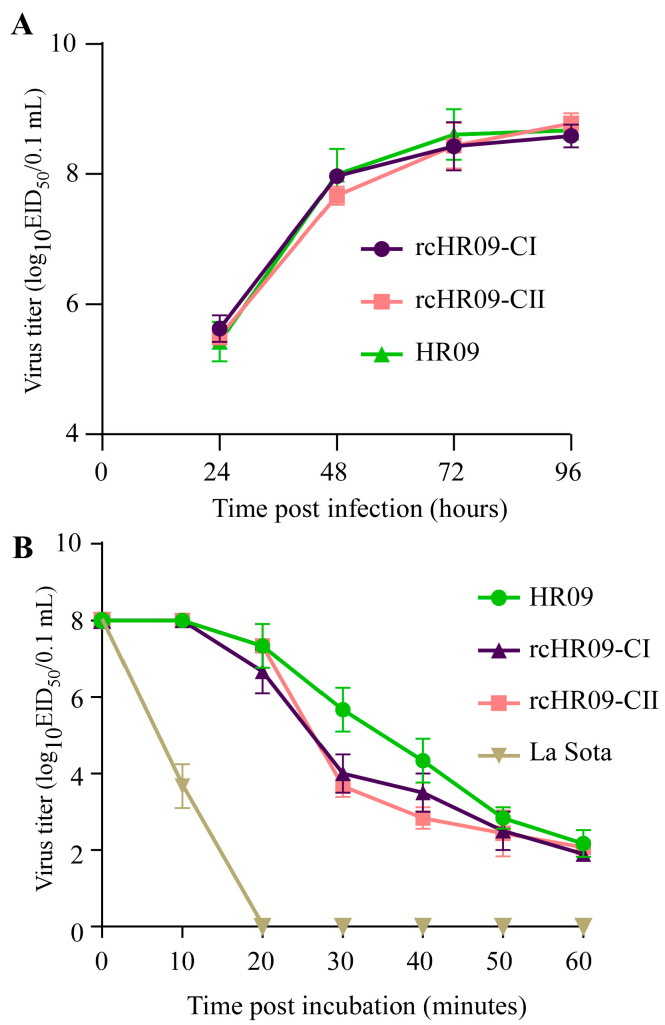
The biological characteristics of the chimeric NDV rcHR09-CI and rcHR09-CII. (**A**) The two chimeric NDV rcHR09-CI and rcHR09-CII have similar growth kinetic with their maternal strain HR09. The growth curve of the two chimeric viruses and their maternal strain HR09 were measured with EID_50_ method in 10-day-old SPF embryonated chicken eggs. (**B**) The two chimeric NDV rcHR09-CI and rcHR09-CII share the thermostability with HR09. Total 10^8^ EID_50_ of the NDV rcHR09-CI, rcHR09-CII, HR09, and La Sota strains were incubated in 56 °C for 10-, 20-, 30-, 40-, 50-, and 60-min, respectively. Then, the titers of the four NDVs were measured using EID_50_ method.

To check whether the replacement of F gene affects the replication of NDV, growth kinetics were performed in 10-day-old embryonated chicken eggs by measuring the EID_50_ of them. The NDVs were diluted and incubated in 10-day-old embryonated chicken eggs, and the allantoic fluid was collected at 24-, 48-, 72-, and 96-h post incubation. The EID_50_ results of them showed that both the rescued chimeric rcHR09-CI and rcHR09-CII shared a similar growth kinetics with their parental HR09 strain (Figure 3A), which demonstrated that the replacement of F gene had no effect to the replication of HR09.

As HR09 strain was a thermostable NDV strain, whether the replacement of F protein affected its thermostability was also investigated. Each chimeric virus and HR09 with same virus titer (10^8^ EID_50_) were treated with 10-, 20-, 30-, 40-, 50-, and 60-min under 56 °C. Then, the virus titer of them was measured by performing EID_50_ assay. The results showed that the two chimeric viruses and their parental strain HR09 could keep infectious for more than 60 min, while the non-thermostable La Sota strain just remained infectious for no more than 20 min (Figure 3B). These data indicated that the chimeric viruses rcHR09-CI and rcHR09- 218 CII were still thermostable.

In this study, the F gene of VIII HR09 were replaced with mutated F gene of VII DT-2014 to get two attenuated chimeric NDVs. To evaluate the virulence of chimeric rcHR09-CI and rcHR09-CII, the MDT and ICPI values were detected. The results showed that the MDT value of all the two chimeric NDVs was larger than 120 h. As control, the MDT value of their parental HR09 strain was 57.6 h. The ICPI values of rcHR09-CI and rcHR09-CII were zero, as control, the ICPI of HR09 was 1.8 (Table 1). The virulence of NDV based on MDT value has been classified into three groups: velogenic (under 60 h), mesogenic (60–90 h), and lentogenic (more than 90 h). Meanwhile, ICPI value is also a common criterion to determine the virulence of NDV: velogenic (under 60 h), mesogenic (60–90 h), and lentogenic (more than 90 h): velogenic (1.5–2.0), mesogenic (0.7–1.5), and lentogenic (0.0–0.7) [24]. Collectively, these data demonstrate that both the rescued chimeric rcHR09-CI and rcHR09-CII were highly attenuated by altering the cleavage sites of F protein.

In addition, the stability of the two chimeric NDVs was detected by incubating for 20 passages in 10-day-old embryonated chicken eggs, and the passage 10 and 20 were chosen to measure the MDT and ICPI values. The results showed that the MDT values of passage 10th and passage 20th were all larger than 120 h, and the ICPI values of passage 10th and passage 20th were all zero (Table 1). These data showed that after 20 passages in 10-day-old embryonated chicken eggs, the MDT and ICPI values of two chimeric NDVs had no significant difference, which demonstrated that the two rescued chimeric NDVs were relatively stable.

### 3.4. A High HI Antibody Titer Was Induced after Immunization of the Chimeric NDVs

**Figure 4:**
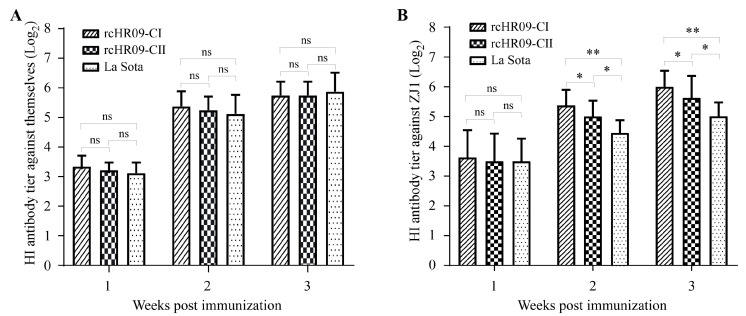
The chimeric viruses induced a high level of antibody response. The SPF chickens were immunized with 10^6^ EID_50_ rcHR09-CI, rcHR09-CII and the commercial vaccine La Sota strain through oculonasal route, respectively. The serum was collected at 1-, 2-, and 3-weeks post immunization, the antibody titer was measured using HI method against themselves (**A**) and the cross HI antibody titer against ZJ1 (**B**). (ns: no significant difference; * *p* < 0.05; ** *p* < 0.01).

To check whether the immunization of the rescued chimeric NDVs could induce the antibody response in chickens, 48 8-day-old SPF chickens were divided into four groups. Different groups were inoculated with 10^6^ EID_50_ of rcHR09-CI, rcHR09-CII, La Sota strain, or 100 μL DMEM via the oculonasal route, respectively. The serum of immunized chickens was collected at 7-, 14-, and 21-days post immunization (dpi) for HI antibody titer determination. The HI antibody of collected serum against the immunized NDV strain showed that there was no significant difference among the serum of rcHR09-CI, rcHR09-CII, and La Sota in 7 dpi. Moreover, with time going on, the HI antibody titer was growing in serum of 14 and 21 dpi, and there was no significant difference between three groups (Figure 4A).

In addition, the cross-reactive HI antibody titer against ZJ1 strain were detected in order to elevate the immunization effects of chimeric NDVs against genotype VII NDV. The results showed that there was no significant difference between three NDVs immunized groups in 7 dpi, and the HI antibody titer was growing with time going on. Different from the HI antibody titer against immunized strain, the HI antibody titers against ZJ1 strain of rcHR09-CI- and rcHR09-CII-immunized groups were significantly higher than the La Sota vaccine immunized group, and rcHR09-CI group induced the highest HI antibody titer among these three groups (Figure 4B).

### 3.5. Viral Shedding Was Reduced When the Immunized Chickens Were Challenged with Virulent Genotype VII ZJ1 Strain 

**Figure 5:**
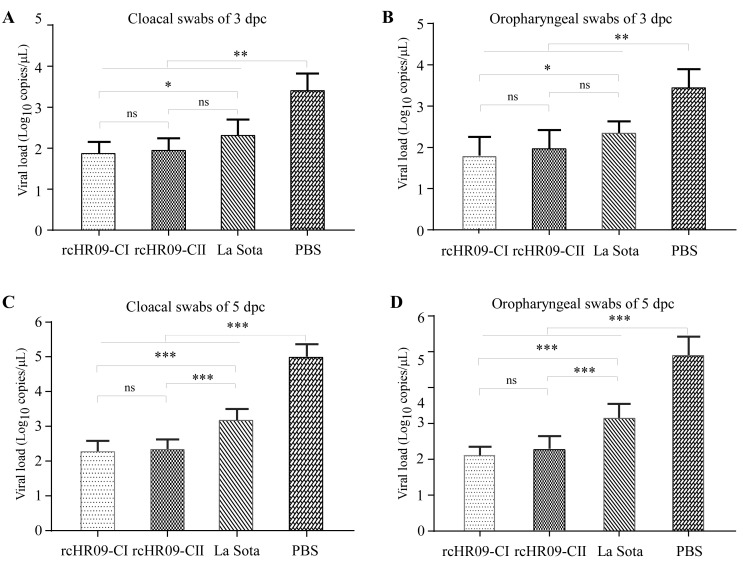
QRT-PCR quantitation of NDV ZJ1 shedding in challenged chickens. The chickens were challenged with 10^6^ EID_50_ NDV genotype VII ZJ1 strain at 21 days post immunization. The cloacal swab samples (**A**,**C**) and oropharyngeal swab samples (**B**,**D**) were collected at the 3- and 5-days post challenge, and the levels of NDV ZJ viral shedding was quantitated via QRT-PCR. Values were shown as mean values ± standard deviation (mean ± SD) in each group. (ns: no significant difference; * *p* < 0.05; ** *p* < 0.01; *** *p* < 0.001).

In this study, the immunized SPF chickens were challenged with genotype VII ZJ1 strain to evaluate the protective effect of chimeric viruses against genotype VII NDV. At 21 dpi, the immunized chickens were challenged with 10^5^ EID_50_ ZJ1 strain, and the cloacal and oropharyngeal swabs from all the challenged chickens of each group were collected at the 3- and 5-days post challenge (dpc) for viral shedding detection. The results showed that the immunization of rcHR09-CI, rcHR09-CII, and La Sota groups could reduce the viral shedding in cloacal swabs (Figure 5A,C) and oropharyngeal swabs at the 3- and 5-dpc (Figure 5B,D) compared with control group. Compared with La Sota group, the viral shedding in two chimeric NDVs was significantly lower than that in La Sota immunized groups, which demonstrated that the two chimeric NDVs had a better protective efficiency than La Sota strain against the challenge of genotype VII NDV.

### 3.6. The Two Chimeric NDVs Provided Full Protection against the Challenge of Genotype VII ZJ1 Strain

**Figure 6:**
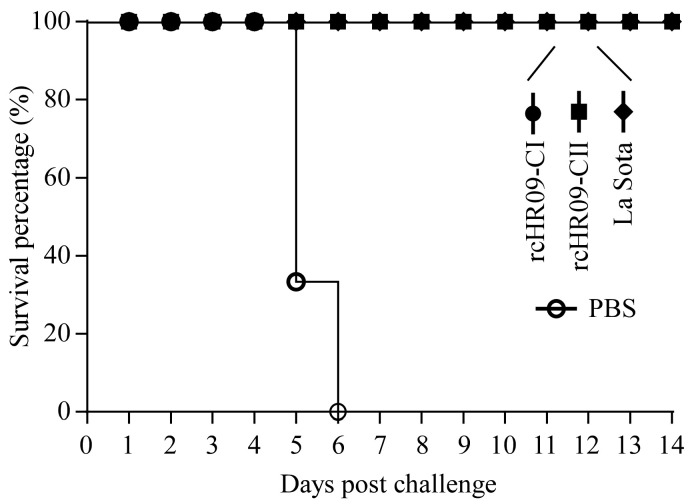
Chimeric NDV rcHR09-CI and rcHR09-CII provide full protection against the challenge of ZJ1. At 3 weeks post immunization, the chickens were challenged with 10^6^ EID_50_ ZJ1 strain. The survival status was monitored for a total of 14 days.

Because the challenge of genotype VII ZJ1 strain can lead to lethal consequences, except the detection of viral shedding, the death of challenged chickens was also recorded every day until 14 days. The results showed that all chickens in control group showed clinical signs of spiritual depression, respiratory disorder, diarrhea, and died at 5- to 6-dpc, and there were no clinical signs and no chickens dead in rcHR09-CI, rcHR09-CII, and La Sota groups until 14 dpc (Figure 6), which demonstrated that all chimeric NDVs and La Sota vaccine strain could provide full protection against the challenge of genotype VII NDV.

## 4. Discussion

Despite multiple kinds of NDV vaccines being used in the world, the ND still happens in many places of the world. One problem might be the genotype of vaccine strain is not match with the prevalent strain. These genotype-mismatched vaccines can provide some protection against the challenge of virulent NDVs; however, they cannot protect the chickens from infection, and cannot effectively reduce the viral shedding [25,26]. The class II genotype VII NDV is a major genotype prevalent in the Middle East, Africa, and Asia regions; however, the vaccines used in these places are usually genotype Ⅱ La Sota and B1 strains, which are not the same genotype with prevalent strains [27,28,29]. In this study, in order to generate a thermostable genotype VII-matched vaccine candidate, a thermostable genotype VIII HR09 strain was used as the backbone, and two chimeric genotype VII-matched vaccine candidates were constructed to fulfill the need for a vaccine for genotype VII.

Until now, multiple thermostable NDV vaccine candidates have been developed. The first thermostable NDV V4 was isolated from the proventriculus of chickens in 1966 [30]. V4 was a natural lentogenic strain, and phylogenetic analysis results showed that it was belonged to the genotype Ⅱ NDV. Different from V4, the thermostable strain HR09 used in our study was belonged to a virulent strain [14]. If we want to put it into clinical use, the first thing to do is determine how to make it as an avirulent strain. Thanks to the development of reverse genetic system, the construction of avirulent genotype-matched NDV vaccine candidate has achieved significant progress. As the cleavage site of F protein has been demonstrated to be the major virulence determinant of NDV [31], a simple way to generate the attenuated NDV is replacing the cleavage site of F protein in virulent strain to that of avirulent strain. Meanwhile, it has been reported that the F protein is an important immunogenetic protein that could induce the neutralization antibody to protect the immunized chickens against the challenge of virulent NDV [32,33,34]. In addition, it was reported that the cleavage site of NDV could affect the immune response in immunized chickens [11]. In this study, we replaced the F gene of a thermostable HR09 with the F gene of NDV genotype VII virulent DT-2014 strain; meanwhile, the cleavage sites of DT-2014 were also mutated to it of the avirulent NDV strains of class Ι and class Ⅱ, respectively (Figure 1). As expected, the virulence determination results showed that both of the two chimeric thermostable NDVs are highly attenuated (Table 1). Meanwhile, it was also found that both of the two chimeric NDVs showed similar growth kinetics to their parental HR09 strain (Figure 3A). As the HN protein was the major protein that was responsible for the thermostability of HR09, the two chimeric NDVs still kept the thermostability just like their parental HR09 strain (Figure 3B).

Multiple studies have reported that genotype-matched NDV vaccine can induce a higher level of humoral immunity response, and is more efficient in reducing the viral shedding than heterologous vaccine, despite both genotype-matched and heterologous vaccines can protect the infected chickens from obvious clinical signs and death [35,36,37]. In this study, the HI antibody titer was measured at 7-, 14-, and 21-dpi, the results of antibody titer against the immunized train showed that there was no significance different among rcHR09-CI, rcHR09-CII, and La Sota strains (Figure 4A), while the HI antibody against the genotype VII ZJ1 strain showed that the two thermostable chimeric NDVs induced a significant higher level than La Sota strain, and among all of three immunized NDVs, rcHR09-CI induced the highest level of HI antibody (Figure 4B). This result was consistent with previous report that the cleavage site of F protein could affect the immunity response of vaccine candidate in chickens. The genotype VII ZJ1 challenge experiment showed that all of the three immunized groups showed a significant reduction in viral shedding compared with control group, and the two chimeric thermostable NDVs could reduce the viral shedding more efficiently than La Sota strain (Figure 5). The survival curve showed that all rcHR09-CI, rcHR09-CII, and La Sota strains could provide complete protection against the challenge of ZJ1 strain (Figure 6). Combined, these data showed that the two genotype VII-matched chimeric NDVs constructed in this study could provide efficient protection against the challenge of genotype VII ZJ1 strain, and could reduce the shedding of virus more efficiently than La Sota strain.

In summary, this study constructed two thermostable chimeric NDV vaccine candidates, which utilize thermostable genotype VIII NDV virulent HR09 strain as the backbone and replaced its F gene with that of the genotype VII DT-2014 strain. Meanwhile, the F protein cleavage site of DT-2014 was mutated from ^112^RRQRR↓F^117^ to ^112^ERQER↓L^117^ and ^112^GRQGR↓L^117^, respectively. The two chimeric NDVs preserve the thermostability of HR09, and the virulence tests by MDT and ICPI indicate that they are successfully attenuated. Both of the chimeric NDVs could induce a high level of antibody response, significantly reduce the viral shedding, and provide complete protection upon the challenge of virulent genotype VII ZJ1 strain.

## Figures and Tables

**Table 1:** The pathogenicity and stability evaluation of the two chimeric NDVs (total 20 passages).

Strain	Passage 1		Passage 10		Passage 20	
	MDT	ICPI	MDT	ICPI	MDT	ICPI
rcHR09-CI	≥120	0	≥120	0	≥120	0
rcHR09-CII	≥120	0	≥120	0	≥120	0
HR09	57.6	1.8	58	1.8	57	1.8

## Data Availability

Not applicable.
